# Identification of H_2_S_3_ and H_2_S produced by 3-mercaptopyruvate sulfurtransferase in the brain

**DOI:** 10.1038/srep14774

**Published:** 2015-10-06

**Authors:** Yuka Kimura, Yukiko Toyofuku, Shin Koike, Norihiro Shibuya, Noriyuki Nagahara, David Lefer, Yuki Ogasawara, Hideo Kimura

**Affiliations:** 1 Department of Molecular Pharmacology, National Institute of Neuroscience, National Center of Neurology and Psychiatry, 4-1-1 Ogawahigashi, Kodaira, Tokyo 187-8502, Japan; 2 Department of Analytical Biochemistry, Meiji Pharmaceutical University, 2-522-1 Noshio, Kiyose, Tokyo 204-8588, Japan.; 3 Radioisotope Center, Nippon Medical School, 1-1-5 Sendagi, Bunkyo, Tokyo 113-8602, Japan.; 4 Department of Pharmacology and Experimental Therapeutics and Cardiovascular Center of Excellence, LSU Health Science Center, New Orleans, LA 70112, USA

## Abstract

Hydrogen polysulfides (H_2_S_n_) have a higher number of sulfane sulfur atoms than hydrogen sulfide (H_2_S), which has various physiological roles. We recently found H_2_S_n_ in the brain. H_2_S_n_ induced some responses previously attributed to H_2_S but with much greater potency than H_2_S. However, the number of sulfur atoms in H_2_S_n_ and its producing enzyme were unknown. Here, we detected H_2_S_3_ and H_2_S, which were produced from 3-mercaptopyruvate (3 MP) by 3-mercaptopyruvate sulfurtransferase (3MST), in the brain. High performance liquid chromatography with fluorescence detection (LC-FL) and tandem mass spectrometry (LC-MS/MS) analyses showed that H_2_S_3_ and H_2_S were produced from 3 MP in the brain cells of wild-type mice but not 3MST knockout (3MST-KO) mice. Purified recombinant 3MST and lysates of COS cells expressing 3MST produced H_2_S_3_ from 3 MP, while those expressing defective 3MST mutants did not. H_2_S_3_ was localized in the cytosol of cells. H_2_S_3_ was also produced from H_2_S by 3MST and rhodanese. H_2_S_2_ was identified as a minor H_2_S_n_, and 3 MP did not affect the H_2_S_5_ level. The present study provides new insights into the physiology of H_2_S_3_ and H_2_S, as well as novel therapeutic targets for diseases in which these molecules are involved.

Hydrogen polysulfides (H_2_S_n_) have a higher number of sulfane sulfur atoms than hydrogen sulfide (H_2_S), which has various physiological roles including neuromodulation, vascular tone regulation, and cytoprotection against ischemic insults^1^,^2^,^3^,^4^,^5^,^6^,^7^. Only the bound form of polysulfides, which bridges cysteine residues in proteins, has been observed *in vitro*^8^,^9^; 3-mercaptopyruvate (3 MP) is reported to be a substrate for its enzymatic production^10^. However, endogenous diffusible polysulfides (H_2_S_n_) were not known to exist. We recently found H_2_S_n_ in the brain and also showed that they activated transient receptor potential ankyrin 1 (TRPA1) channels approximately 300 times more potently than did hydrogen sulfide (H_2_S)^11^,^12^,^13^. Anxiety-related behavior was observed in both 3-mercaptopyruvate sulfurtransferase knockout (3MST-KO)^14^ and TRPA1-KO mice^15^ as well as mice in which TRPA1 channels are pharmacologically inhibited^15^, suggesting the involvement of TRPA1 channels and 3MST in the induction of anxiety-like behavior. H_2_S_n_ also facilitates the translocation of nuclear factor-like 2 (Nrf2) to the nucleus by modifying its binding partner kelch-like ECH-associated protein 1 (Keap1)^16^, regulates the activity of the tumor suppressor phosphatase and tensin homolog (PTEN)^17^, and reduces blood pressure by dilating vascular smooth muscle^18^. Despite these important physiological effects, its producing enzyme and the number of sulfur atoms in H_2_S_n_ were unknown. Here, we identified H_2_S_3_ as an important H_2_S_n_ and 3MST as its producing enzyme. H_2_S_3_ was also produced from H_2_S by 3MST and rhodanese.

## Results

### Production of H_2_S_3_ and H_2_S from 3 MP by 3MST

**Figure 1:**
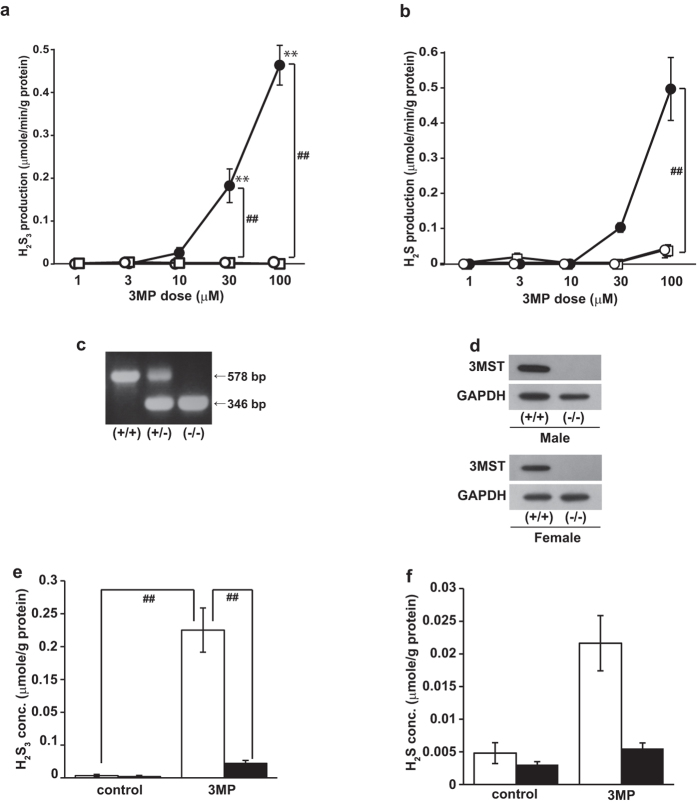
H_2_S_3_ and H_2_S are produced by 3MST from 3 MP. (**a,b**) The production of H_2_S_3_ (**a**) and H_2_S (**b**) from 3 MP with lysates of COS cells expressing 3MST (●), rhodanese (○), or empty vector (○) as a source of the enzymes incubated for 15 min. (**c,d**) Determination of the 3MST genotype by polymerase chain reaction (PCR) (**c**). Western blot analysis (**d**) of 3MST in the brains of wild-type (+/+) and 3MST-KO (−/−) mice. (+/−): heterozygote. GAPDH was used as a control. (**e,f**) Concentrations of H_2_S_3_ (**e**) and H_2_S (**f**) in whole cells prepared from wild-type (open bar) and 3MST-KO-mice (filled bar). Suspensions of brain cells were exposed to 500 μM 3 MP (distilled water for a control) for 15 min (the intracellular 3 MP concentration reached 0.40 ± 0.09 μmol/g protein), and the concentrations of H_2_S_3_ (**e**) and H_2_S (**f**) were measured as monobromobimane adducts from cell lysates. ** and ##p < 0.01. **: the comparison with a value at 1 mM for (**a,b**). All data represent the mean ± standard error of the mean (SEM) of at least three experiments.

Because 3 MP, a substrate of 3MST, produces polysulfides, which bridge cysteine residues in the proteins^10^,^19^, and cells expressing 3MST contain greater amounts of the form of polysulfides than do controls^6^,^19^, we hypothesized that 3MST may also produce diffusible polysulfides (H_2_S_n_). To address this problem, we examined H_2_S_n_ production using lysates of COS cells expressing 3MST as a source of the enzyme. There are various methods to measure H_2_S including colorimetric methylene blue method, ion-selective or polarographic electrodes, gas chromatography, and monobromobimane assay^20^. The variations of endogenous levels of H_2_S in tissue and blood samples have been reported and are largely attributed to the contaminant of H_2_S released from acid-labile sulfur or bound sulfane sulfur mostly caused during the preparation of the samples rather than the methods^21^. The monobromobimane analysis with high performance liquid chromatography with fluorescence detection (LC-FL) was previously applied to the quantitative analysis of H_2_S_n_^13^, and also to the analysis of H_2_S using both LC-FL and mass spectrometry^22^. In the present study LC-FL and LC-tandem mass spectrometry (LC-MS/MS) was used to analyze monobromobimane adducts of H_2_S_n_ and H_2_S. H_2_S_3_ and H_2_S were produced from 3 MP by 3MST in a concentration-dependent manner (Fig. 1a,b and Supplementary Fig. 1). Rhodanese, which is homologous to 3MST and may produce the bound form of polysulfides^10^, generated neither H_2_S_3_ nor H_2_S from 3 MP (Fig. 1a,b). Lysates of cells transfected with an empty vector did not produce either molecule (Fig. 1a,b). These observations suggest that 3MST produces H_2_S_3_ (as a major H_2_S_n_) and H_2_S from 3 MP.

**Figure 2:**
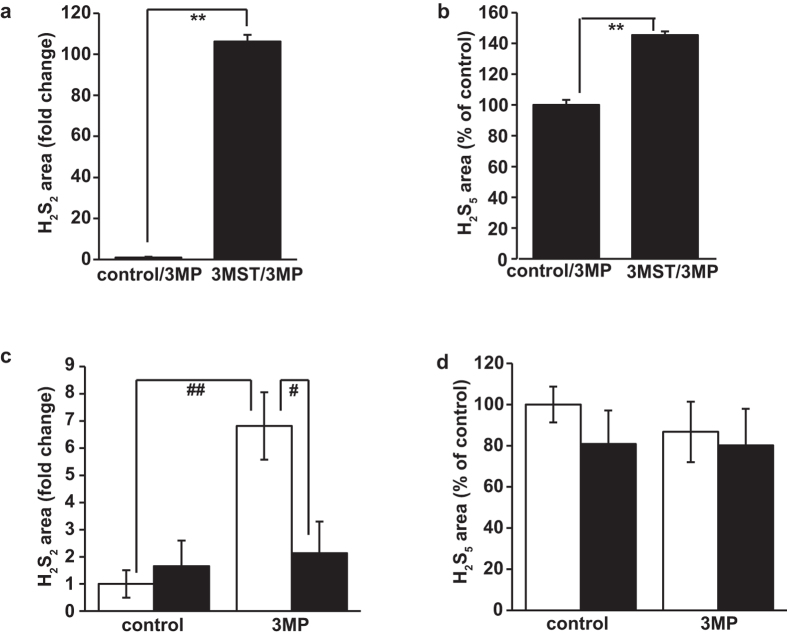
Production of H_2_S_2_ and H_2_S_5_ from 3 MP by 3MST. (**a,b**) Production of H_2_S_2_ (**a**) and H_2_S_5_ (**b**) from 3 MP with lysates of COS cells expressing 3MST as a source of the enzyme. Control: lysates of cells transfected with an empty vector. (**c,d**) Production of H_2_S_2_ (**c**) and H_2_S_5_ (**d**) in whole cells prepared from brains of wild-type (open bar) and 3MST-KO mice (filled bar) exposed to 500 μM 3 MP (distilled water for a control) for 15 min. A H_2_S_5_ standard was not available, and H_2_S_2_ was detected by LC-MS/MS but not clearly recognized by LC-FL in which the H_2_S_2_ peak was buried within the H_2_S_5_ peak. For these reasons, relative values are shown for H_2_S_2_ and H_2_S_5_. ** and ##p < 0.01. All data represent the mean ± standard error of the mean (SEM) of at least three experiments.

H_2_S_2_ was detected as a minor product, and H_2_S_5_ increased approximately 40% from its basal level in the presence of 3 MP (Fig. 2a,b). The relative levels for H_2_S_2_ and H_2_S_5_ are shown (Fig. 2a,b), as a H_2_S_5_ standard was not available and H_2_S_2_ was detected by LC-MS/MS but not clearly recognized by LC-FL, in which the H_2_S_2_ peak was buried within the H_2_S_5_ peak.

### Production of H_2_S_3_ and H_2_S in whole cells

We examined whether whole cells were able to produce H_2_S_3_ and H_2_S following exposure to 3 MP. Suspensions of brain cells prepared from wild-type and 3MST-KO mice were used (Fig. 1c–f). To provide 3 MP intracellularly, whole cells prepared from wild-type mice were exposed to 3 MP. Wild-type cells produced significantly more H_2_S_3_ (0.23 ± 0.03 μmol/g protein; ~65× increase compared to controls) and H_2_S (0.02 ± 0.00 μmol/g protein; ~5× increase compared to controls). Conversely, H_2_S_3_ and H_2_S production did not significantly increase in cells obtained from 3MST-KO mice following 3 MP exposure (Fig. 1e,f). These observations suggest that H_2_S_3_ is produced not only in *in vitro* enzymatic reactions, but also in whole cells. Moreover, the concentration of H_2_S_3_ was approximately 10 times greater than that of H_2_S in whole cells. The level of H_2_S_2_ was also increased in the presence of 3 MP, while that of H_2_S_5_ was not significantly changed regardless of the presence or absence of 3 MP (Fig. 2c,d).

H_2_S_3_ was detected in cells prepared from wild-type mice even in the absence of 3 MP exposure, suggesting the presence of endogenous H_2_S_3_ (3.4 ± 2.2 nmol/g protein in the brain). H_2_S was also detected at a level of 4.8 ± 1.6 nmol/g protein (Fig. 1e,f). The endogenous concentrations of H_2_S_3_ and H_2_S were lower in 3MST-KO mice, 1.9 ± 1.9 and 3.0 ± 0.4 nmol/g protein, respectively (Fig. 1e,f), but these values did significantly differ from those found in wild-type mice.

### H_2_S_3_ and H_2_S production depends on 3MST catalytic site

**Figure 3:**
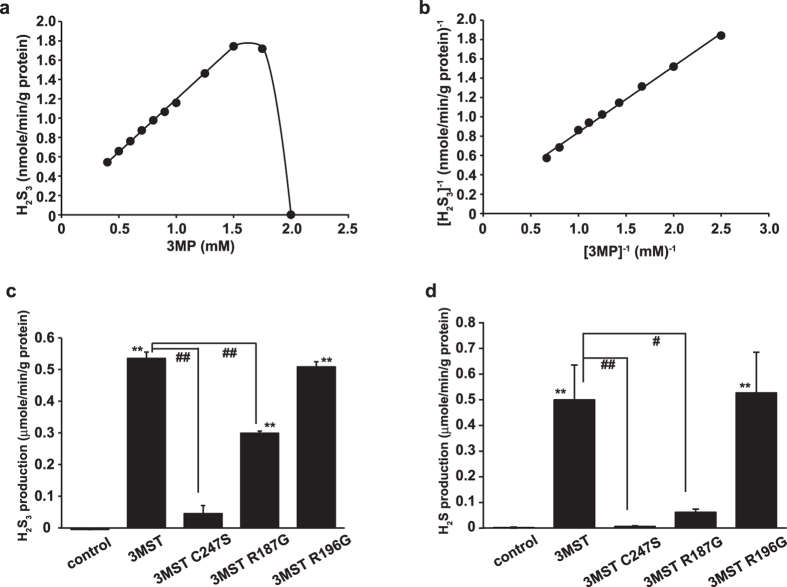
H_2_S_3_ and H_2_S production depends on 3MST catalytic site. (**a,b**) The kinetics of H_2_S_3_ generation by 3MST in the presence of 3 MP. The production of H_2_S_3_ from 3 MP by purified 3MST (**a**), and its Lineweaver-Burk plot (**b**). (**c,d**) Production of H_2_S_3_ (**c**) and H_2_S (**d**) from 3 MP by various defective 3MST mutants. Lysates of COS cells expressing 3MST mutants as a source of the enzymes were incubated with 100 μM 3 MP for 15 min, and the H_2_S_3_ (**c**) and H_2_S (**d**) produced were measured. Control: lysates of cells transfected with an empty vector. #p < 0.05, ** and ##p < 0.01. **: the comparison with a value of an empty vector for (**c,d**). All data represent the mean ± standard error of the mean (SEM) of at least three experiments.

We examined the affinity of 3MST for 3 MP using purified recombinant 3MST produced by bacteria (Fig. 3a,b). 3MST produced H_2_S_3_ from 3 MP in a concentration-dependent manner up to 1.5 mM. The K_m_ value of 3MST for 3 MP was 4.5 ± 1.5 mM.

To confirm the production of H_2_S_3_ and H_2_S by 3MST, the production of both molecules by defective 3MST mutants was compared with that of wild-type 3MST using lysates of COS cells expressing the mutant or wild-type 3MST as a source of the enzymes^23^,^24^. Introduction of the mutant, in which the active site cysteine 247 was replaced with serine (C274S), resulted in greatly decreased production of H_2_S_3_ and H_2_S to 8.5% and 1.2%, respectively, of that produced in the presence of wild-type 3MST (Fig. 3c,d). The R187G mutant produced H_2_S_3_ and H_2_S at 55.9% and 12.2%, respectively, of that produced by wild-type 3MST; R196G produced H_2_S_3_ and H_2_S at 94.1% and 105.5%, respectively, of that produced by the wild-type (Fig. 3c,d). These observations suggest that the production of H_2_S_3_ and H_2_S greatly depends on the 3MST catalytic site, C247.

### H_2_S_3_ is localized in the cytosol

**Figure 4:**
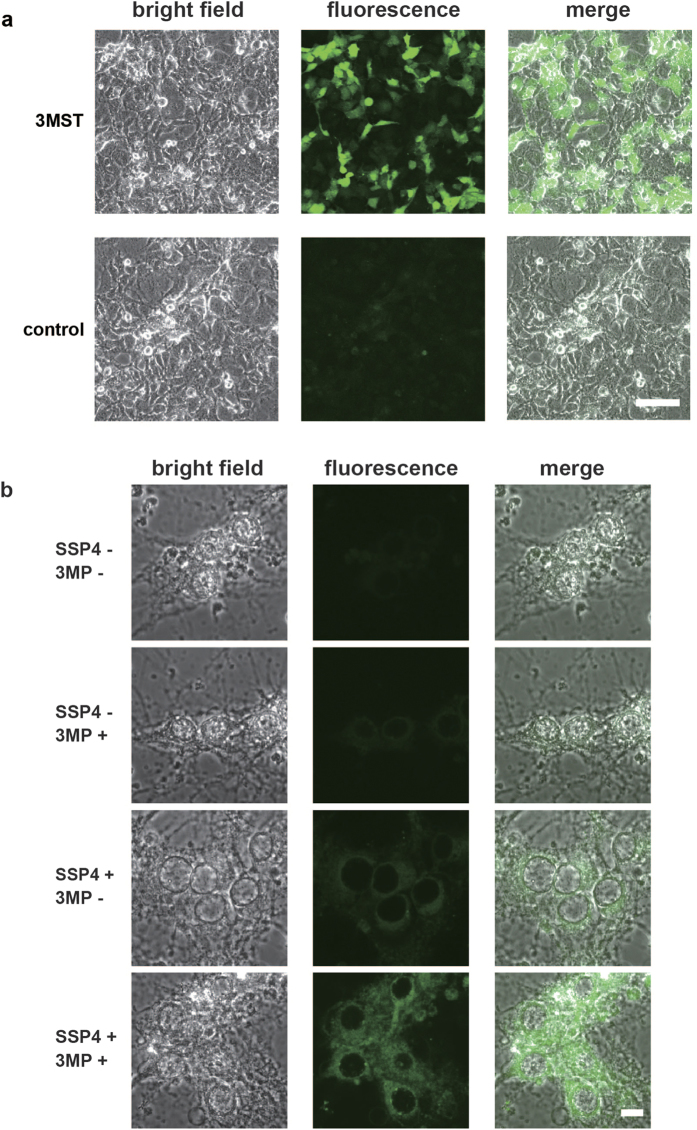
Cellular localization of H_2_S_3_ produced from 3 MP in COS cells expressing 3MST and in primary neuronal cultures. (**a**) Localization of H_2_S_3_ in COS cells expressing 3MST. Forty-eight hours after transfection with the 3MST cDNA expression plasmid, COS cells were incubated with 50 μM SSP4 for 20 min, and then exposed to 500 μM 3 MP for 10 min. Cells transfected with an empty vector were used as controls. Scale bar = 100 μm. (**b**) Localization of H_2_S_3_ in primary neuronal cultures. Primary neuronal cultures were incubated with 50 μM SSP4, and then exposed to 500 μM 3 MP for 10 min. Scale bar = 10 μm.

We examined the cellular localization of H_2_S_3_ produced by 3MST by using COS cells expressing 3MST and loaded with SSP4, a polysulfide-sensitive fluorescence probe^25^. H_2_S_3_ produced de novo from 3 MP incorporated into cells was localized in the cytosol (Fig. 4a). H_2_S_3_ was also localized in the cytosol in primary neuronal cultures (Fig. 4b).

### H_2_S_3_ production from H_2_S by 3MST

**Figure 5:**
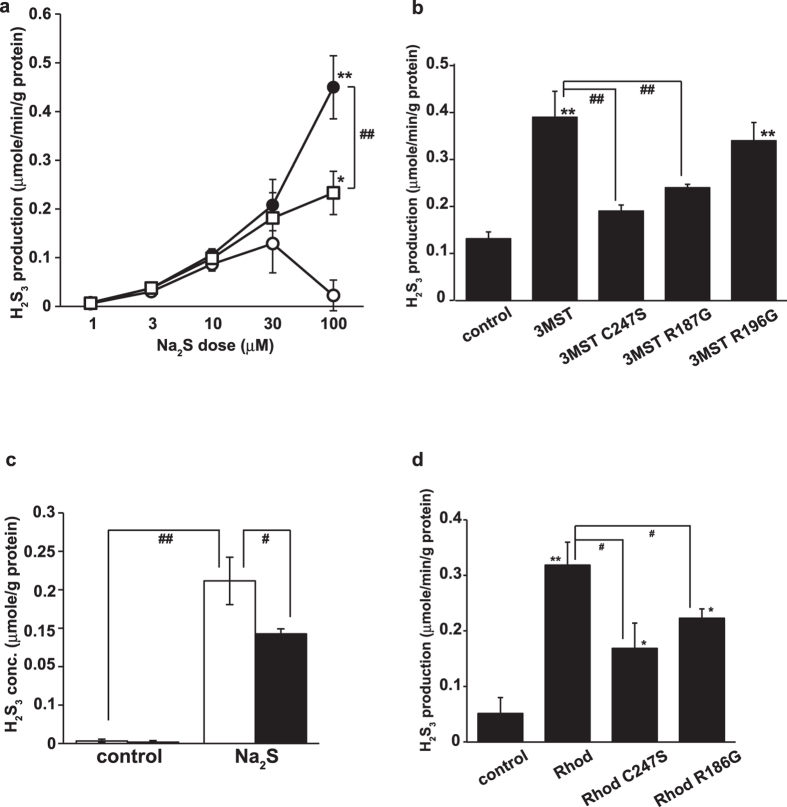
Production of H_2_S_3_ from H_2_S by 3MST and rhodanese. (**a**) Production of H_2_S_3_ from H_2_S by 3MST or rhodanese. Lysates of COS cells expressing 3MST (●), rhodanese (○), or empty vector (○) as a source of the enzymes were incubated with Na_2_S for 15 min. (**b**) The production of H_2_S_3_ from H_2_S by 3MST mutants. The production of H_2_S_3_ from 100 μM Na_2_S in lysates of COS cells expressing various defective 3MST mutants was compared with that of wild-type 3MST. Control: lysates of cells transfected with an empty vector. (**c**) Production of H_2_S_3_ from H_2_S in whole cells. Suspensions of brain cells prepared from wild-type and 3MST-KO mice were exposed to 500 μM Na_2_S for 15 min (the intracellular H_2_S concentration reached 0.11 ± 0.08 μmol/g protein), and H_2_S_3_ levels in the cells were analyzed. Distilled water was applied as a control. (**d**) The production of H_2_S_3_ from H_2_S by rhodanese mutants. The production of H_2_S_3_ from 100 μM Na_2_S by lysates of COS cells expressing various defective rhodanese mutants as a source of the enzymes was compared with that of wild-type rhodanese. Control: lysates of cells transfected with an empty vector. The amounts of H_2_S_n_ species produced by the oxidation of Na_2_S in the absence of cells or cell lysates were subtracted. * and #p < 0.05, ** and ##p < 0.01. * and **: the comparison with a value at 1 μM Na_2_S for (**a**), and with that of an empty vector for (**b,d**). All data represent the mean ± SEM of at least three experiments.

Because H_2_S_n_ can also be generated from H_2_S^26^, it is possible that 3MST is involved in its generation. We examined this problem using lysates of COS cells expressing 3MST. Although H_2_S was oxidized to generate H_2_S_3_, even in the absence of 3MST, 3MST significantly accelerated the production of H_2_S_3_ from Na_2_S, a sodium salt of H_2_S; control lysates did not generate H_2_S_3_ (Fig. 5a).

To confirm the production of H_2_S_3_ from 100 μM H_2_S by 3MST, the activity of defective 3MST mutants was compared with that of wild-type 3MST by using lysates of COS cells expressing these mutants. In the case of the C247S mutant, the production of H_2_S_3_ from H_2_S was 48.8% lower than that seen for wild-type 3MST (Fig. 5b). Mutations at the modulatory sites R187G and R196G resulted in 61.5% and 87.2% production of H_2_S_3_, respectively, compared to that produced by wild-type 3MST. These observations suggest that the production of H_2_S_3_ from H_2_S depends on the activity of 3MST.

### H_2_S_3_ production from H_2_S in whole cells

We examined whether H_2_S_3_ was produced from H_2_S in whole cells using brain cell suspensions. Whole cells prepared from wild-type mice and exposed to Na_2_S effectively produced H_2_S_3_; whole cells prepared from 3MST-KO mice produced approximately two-thirds the amount of that produced in wild-type cells (Fig. 5c). These observations suggest that whole cells produce H_2_S_3_ from H_2_S, and that 3MST and another enzyme, which has activity similar to 3MST, are involved in the production.

### H_2_S_3_ production from H_2_S by rhodanese

We examined whether rhodanese was another enzyme able to produce H_2_S_3_ from H_2_S. Lysates of COS cells expressing rhodanese as a source of the enzyme produced H_2_S_3_ from H_2_S in a concentration-dependent manner (Fig. 5a). To confirm this rhodanese-induced production, we examined lysates of COS cells expressing defective rhodanese mutants. In the case of the C247S mutant, H_2_S_3_ production decreased to 52.9% of that seen for wild-type rhodanese (Fig. 5d). A mutation at the R186G modulatory site resulted in production that was decreased to 70.0% of that produced from wild-type rhodanese (Fig. 5d). These observations suggest that rhodanese also produces H_2_S_3_ from H_2_S.

**Figure 6:**
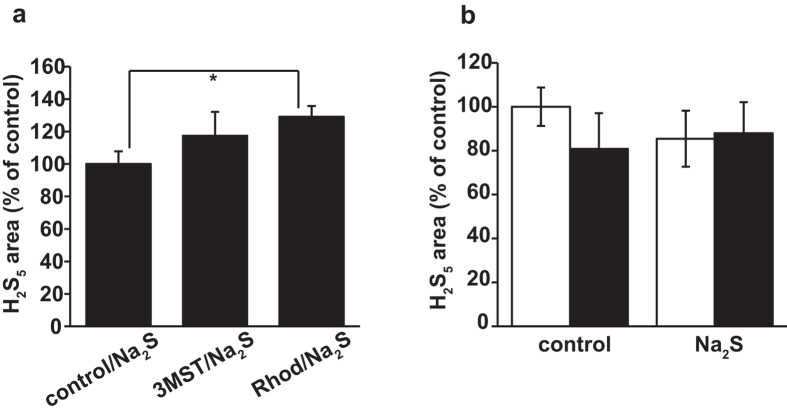
Production of H_2_S_5_ from H_2_S (**a**) H_2_S_5_ production from Na_2_S with lysates of COS cells expressing 3MST or rhodanese as a source of the enzymes. Control: lysates of cells transfected with an empty vector. (**b**) Production of H_2_S_5_ in whole cells prepared from brains of wild-type (open bar) and 3MST-KO mice (filled bar) exposed to 500 μM Na_2_S for 15 min. Distilled water was applied as a control. A H_2_S_5_ standard was not available. For this reasons, relative values are shown for H_2_S_5_. *p < 0.05. All data represent the mean ± standard error of the mean (SEM) of at least three experiments.

The production of H_2_S_5_ from H_2_S slightly increased in the case of enzymatic production with lysates of COS cells expressing rhodanese, but it did not significantly change in brain cell suspensions, even in the presence of H_2_S (Fig. 6a,b).

## Discussion

The present study identified H_2_S_3_ as an important H_2_S_n_ species in the brain, and further identified 3MST as the enzyme responsible for H_2_S_3_ production. Although the basal endogenous concentration of H_2_S_3_ in cells was found to be similar to that of H_2_S, the H_2_S_3_ concentration dramatically increased with increased intracellular levels of 3 MP in the cells of wild-type mice (Fig. 1e,f). This increase in H_2_S_3_ concentration was not observed in cells of 3MST-KO mice (Fig. 1e,f). The H_2_S_3_ and H_2_S concentrations increased by 65× and 5×, respectively, in the cells of wild-type mice. Although H_2_S_2_ was only present as a minor H_2_S_n_ species, H_2_S_2_ levels also increased significantly from basal levels in the presence of 3 MP (Fig. 2c). In contrast to the findings for H_2_S_3_ and H_2_S, H_2_S_5_ levels did not significantly change in cells regardless of the intracellular increase in the concentration of 3 MP and H_2_S_5_ levels were furthermore found not to differ significantly between wild-type and 3MST-KO mice (Fig. 2d). The rapid metabolic turnover of H_2_S_3_ and H_2_S suggests roles for these species as signaling molecules. The production of H_2_S_5_ may be regulated by the unknown metabolic pathway.

The *K*_m_ value of 3MST for 3 MP (4.5 mM) in the present study was larger than the previously reported value (1.2 mM)^23^. This discrepancy may be due to assay differences: in the previous study, the production of pyruvate was measured, while in the present study the production of H_2_S_3_ was measured. In the previous study, experiments were furthermore performed in the presence of an acceptor or reducing agent (2-mercaptoethanol), whereas the experiments reported on here were performed in the absence of a reducing agent, as reducing agents remove H_2_S_n_ from the active site of 3MST before H_2_S_n_ production is complete and also reduce H_2_S_n_ to produce H_2_S. Attributing the discrepancy between the previous findings and those presented here to differences in the assays used is furthermore supported by the observation that, in this study, H_2_S_3_ production was found to be strongly suppressed in the presence of 2 mM 3 MP (Fig. 3a), since excess H_2_S_3_ remained in the active site without being removed by a reducing compound and thus suppressed the progress of the reaction^10^.

Sulfhydration or sulfuration has been defined as a process in which sulfur provided by H_2_S attaches to reactive cysteine residues in target proteins^27^,^28^. Activities of enzymes such as cystathionine synthetase-serine dehydratase, aldehyde oxidase, and adenylate kinase are modified by sulfhydration^29^,^30^,^31^. Sulfhydration by H_2_S in particular has been proposed to regulate the catalytic activity of glyceraldehyde 3-phosphate dehydrogenase (GAPDH)^27^; actin polymerization^27^; the activity of ATP-dependent K^+^ channels^32^, which are involved in vascular smooth muscle relaxation; the activity of protein kinase-like endoplasmic reticulum (ER) kinase for the regulation of ER stress^33^; and the activity of parkin, an E3 ubiquitin ligase that is suppressed in Parkinson’s disease^34^.

Sulfhydration by H_2_S thus seems to play a key role in the regulation of many target proteins; however, a theoretical controversy relating to this observation exists: atoms in the same oxidation state do not exchange electrons that do not result in a redox reaction; however, the oxidation state of the sulfur in H_2_S and that in cysteine residues are both in the −2 oxidation state and therefore cannot react with each other^35^. H_2_S is able to sulfhydrate the cysteine residue, in which the thiol is oxidized to sulfenic acid, as in the case of the glutathionylation reaction^36^.

In contrast, because the oxidation state of the sulfane sulfur in H_2_S_n_ is 0, H_2_S_n_ readily sulfhydrates cysteine residues^11^,^12^,^13^,^16^,^17^,^18^. H_2_S_n_ activates TRPA1 channels by sulfhydrating two cysteine residues at the amino terminus of the channels^13^,^37^ and the species also facilitates the translocation of Nrf2 to the nucleus to upregulate antioxidant genes by sulfhydrating its binding partner Keap1 to release Nrf2^16^. H_2_S_n_ rather than H_2_S enhances the activity of PTEN^17^ and H_2_S_n_ is also involved in the regulation of blood pressure by relaxing vascular smooth muscle^18^. Early studies of sulfhydration^27^,^32^,^33^,^34^ likely measured the reaction of cysteine residues with H_2_S_n_ produced by the oxidation of H_2_S or alternatively oxidized cysteine residues which reacted with H_2_S^35^,^38^.

H_2_S reduces the cysteine disulfide bond of target proteins. For example, H_2_S induces angiogenesis mediated by vascular endothelial growth factor receptor 2 by reducing a disulfide bond located between cysteine 1045 and cysteine 1024^39^.

It is intriguing to decipher the way in which cells regulate the production of H_2_S_n_ and H_2_S and how they deliver these species to the appropriate targets. The present study provides new insights into the biology of H_2_S_n_ and H_2_S, as well as into novel therapeutic approaches to diseases in which these molecules are involved.

## Methods

### Animals

All experiments were approved and conformed to the guidelines set by the Small Animal Welfare Committee of the National Institute of Neuroscience, National Center of Neurology and Psychiatry. C57BL6 mice were purchased from Clea Japan Inc. (Tokyo, Japan), and 3MST-KO mice (Mpst, accession #NM 138670) from Texas A&M Institute for Genomic Medicine (Texas, USA).

### Recombinant 3MST

For recombinant enzymes: 3-MST for the kinetic assay was prepared from fusions with glutathione S-transferase (GST) by the modified method previously reported by Smith and Johnson^40^. Briefly, cDNA constructs of GST fusion proteins were incorporated in pGEX-6p-2 plasmid (GE Healthcare Life Sciences, Little Chalfont, USA) and transformed a bacterial line BL21. Bacteria were cultured in 400 ml M9 medium (6 g Na_2_HPO_4_, 3 g KH_2_PO_4_, 0.5 g NaCl, 1 g NH_4_Cl, 1 ml of 1 M MgSO_4_, 5.6 ml of 2 M glucose, 1 ml of 1% thiamine, 0.1 ml of 1 M CaCl_2_, and 100 μg/ml ampicillin in 1 l distilled water) at 20 °C for 24 hr in a shaker (Takasaki Scientific Instruments Corp. Saitama, Japan). When OD600 was reached to 0.6 ~ 0.8, isopropyl β–D-1-thiogalactopyranoside (IPTG) (Sigma, St. Louis, Missouri, USA) was added to make a final concentration of 0.1 mM and further cultured for 24 hr at 20 °C. Bacteria were collected by a centrifugation at 1,673 × g for 15 min and stored at −80 °C. Bacteria collected from 100 ml culture were lysed in 1 ml lysis buffer consisting of 858 μl PBS, 40 μl 25 × complete protease inhibitor cocktail (Hoffmann-La Roche, Basel, Switzerland) 1 μl 1 M DTT, 50 μl 10 mg/ml lysozyme, 1 μl 1 × 10^4^ U/ml DNA ase I, and 50 μl 20% Triton X on ice for 30 ~ 60 min, and then subjected to sonication. Lysates were centrifuged at 7,000 × g for 10 min by MX-100 (Tomy Seiko, Tokyo, Japan), and the supernatant was applied to GST Spin Trap column (GE Healthcare Life Sciences) and kept it for 10 min at room temperature. The spin column was centrifuged at 735 × g for 1 min and washed twice with 200 μl PBS. A hundred μl PreScission protease (GE Healthcare Life Sciences) solution containing 50 mM Tris (pH 8.0), 100 mM NaCl, 1 mM EDTA, 1 mM DTT was added to the column and incubated for 12 ~ 16 hr at 4 °C, and then 3MST, which had been excised from GST-fusion, was recovered by centrifugation at 735 × g for 1 min at room temperature. DTT was removed by PD spintrap G-25 (GE Healthcare Life Sciences).

### Cell lysates

The activity of enzymes expressed in COS-7 (COS) cells was examined. COS cells were transfected with an expression plasmid encoding 3MST- or rhodanese-cDNA using TransIT-LT1 Transfection Reagent (Mirus Bio, Madison, WI, USA) following the procedure recommended by the manufacturer. After washed twice with PBS in the plates, cells were removed from the plate by scraping twice with each 0.3 ml BHM solution consisting of 0.32 M sucrose, 1 mM EDTA, 10 mM Tris-Cl (pH 7.0) and the complete protease inhibitor cocktail (Roche Applied Science, Upper Bavaria, Germany). The resultant 0.6 ml BHM solution containing cells was sonicated and centrifuged at 1,000 × g for 10 min, and the supernatant was used for measuring the enzyme activity. Fifty μl supernatant was mixed with 40 μl 100 mM KHPO_4_ (pH 7.0) and incubated for 5 min at 37 °C, and then 10 μl substrates such as 3-mercaptopyruvate (3 MP, Sigma-Aldrich), Na_2_S (Wako Pure Cheimcal Industries, Osaka, Japan) or a control H_2_O were added to incubate at 37 °C for 15 min. The resultant reaction mixture was subjected to derivatization with monobromobimane (Life Technologies). The mixture was incubated in the presence of 125 mM CHES (pH 8.4) and 2 mM monobromobimane for 20 min at room temperature, and then acetic acid was added to the final concentration of 3% and incubated 15 min on ice. The resulting reaction mixture was centrifuged at 15,000 × g for 10 min, and the supernatant was analyzed by LC-FL (Waters, Milford, MA, USA) and LC-MS/MS (Shimazu, Kyoto, Japan).

### Suspensions of brain cells

The suspensions of brain cells was prepared by the modified method reported previously by Dutton *et al.*^41^. Briefly, brains of 3MST knockout mice or the wild-type mice were removed at the postnatal day 1 or 2 and submerged in the ice-cold Leiboritz’s L-15 medium (Life Technologies, Waltham Massachusetts, USA). After meninges were removed, brains were chopped to approximately 1 mm cubes with scissors in the medium. The suspended brain cubes were centrifuged at 100 × g, 4 °C for 20 sec to remove medium, and washed once with the medium. The brain cubes were incubated in 10 ml basic medium (3 mg/ml BSA fraction V (Sigma-Aldrich, St. Louis, MO, USA), 14 mM glucose (Sigma), 1.2 mM MgSO_4_ in Ca^2+^ free HBSS (Life Technologies) containing 0.025% trypsin EDTA (Life Technologies) for 15 min at 37 °C, and then 10 ml basic medium containing 6.4 μg/ml DNAse I (Sigma-Aldrich), 0.04 mg/ml Soy Bean Tripsin Inhibitor (SBTI) (Sigma) in HBSS was added and gently mixed. The supernatant was removed after centrifugation at 100 × g for 1 min at room temperature. Two ml basic medium containing 40 μg/ml DNAse I, 0.25 mg/ml SBTI, and 3 mM MgSO_4_ in HBSS was added to the brain cubes and mixed gently up and down with a pipette without making foams for 30 times. After a centrifugation at 100 × g for 1 min, cells were recovered and washed with 2 ml HBSS with Ca^2+^ and Mg^2+^ medium (Wako Pure Chemical Industries) containing 14 mM glucose (Sigma-Aldrich) for 3 times, and then preincubated at 37 °C for 1 hr in a shaker at 100 rpm (Taitec Bio-shaker BR-40LF, Saitama, Japan) before used for experiments.

### Production of polysulfide in whole cells

After preincubation for 1 hr at 37 °C, 300 μl suspensions of brain cells were incubated for 15 min at 37 °C in the presence of 500 μM 3 MP (Sigma-Aldrich), or 500 μM Na_2_S (Wako). After the exposure to 3 MP or Na_2_S the suspensions of brain cells were centrifuged at 100 x  g for 30 sec, and the supernatant was removed. Cells were suspended in 300 μl basic medium containing 14 mM glucose in HBSS with Ca^2+^ and Mg^2+^, and removed the supernatant after centrifugation at 100 × g for 30 sec. This step was repeated three times to wash out 3 MP. Cells were sonicated in BHM solution and centrifuged at 15,000 × g for 10 min at room temperature. The supernatant was incubated in the presence of 125 mM CHES (pH 8.4) and 2 mM monobromobimane for 20 min at room temperature, and then acetic acid was added to the final concentration of 3% and incubated for 15 min on ice. The resulting reaction mixture was centrifuged at 15,000 × g for 10 min, and the supernatant was analyzed by LC-FL and LC-MS/MS. Na_2_S_2_, Na_2_S_3_, and Na_2_S_4_ for standard were obtained from Dojindo (Kumamoto, Japan).

### Primary cultures of neurons

Brains were removed from embryonic day 16 C57BL6 (Clea Japan Inc) mice and meniges were dissected in L15 medium (Life Technologies). The tissue was chopped and digested with 0.25% trypsin (Sigma-Aldrich) and 0.1% DNase I (Sigma-Aldrich) in Ca^2+^/Mg^2+^-free PBS for 15 min at 37 °C. After mechanical dissociation, cells were plated onto poly-D-lysine-coated 35 mm dishes (BD Biosciences, San Jose, CA, USA) and cultured in Neurobasal medium (Life Technologies) supplemented with B27 (Life Technologies) for 2 days at 37 °C in 10% CO_2_. Cells were further cultured in the presence of 5 μM cytosine β–D-arabinofuranoside (AraC, Sigma-Aldrich) for 1 day, and washed once with Neurobasal medium supplemented with B27 to remove AraC. Cells cultured for additional 3–4 days were used for experiments.

### LC-FL analysis

Samples derivatized with monobromobimane (mBB) (Life Technologies) were separated with a Waters Symmetry C18 (ID, 250 × 4.6 mm) column (Waters Corp., Milford, MA, USA) with mobile phase A(0.25% formic acid in H_2_O) and B(0.25% formic acid: methanol = 1:1) with a linear gradient from A:B = 6:4 to 2:8 in 8 min with a flow rate of 0.8 ml/min, and remained with A:B = 2:8 for additional 10 min, and then changed to 100% B in the following 7 min. The monobromobimane adduct was monitored with a scanning fluorescence detector (Waters 2475) with an excitation wavelength of 370 nm and an emission wavelength of 485 nm.

### LC-MS/MS analysis

Samples derivatized with monobromobimane (mBB) (Life Technologies) were analyzed by the triple-quadrupole mass spectrometer coupled to HPLC (Shimazu LCMS-8030). Samples were subjected to a reverse phase Symmetry C18 HPLC column (2.1 × 150 mm, Waters) at the flow rate of 0.8 ml/min. The mobile phase consisted of (A) 0.25% formic acid in water and (B) 0.25% formic acid: methanol = 1:1. Samples were separated by eluting with a gradient: 40% B at 0 min, and 80% B at 8 min and remained it for 10 min. The column oven was maintained at 35 °C. The effluent was subjected to the mass spectrometer using an electrospray ionization (ESI) interface operating in the negative- or positive-ion mode. The source temperature was set at 400 °C, and the ion spray voltage was at 4.5 kV. Nitrogen was used as a nebulizer and drying gas. The tandem mass spectrometer was tuned in the multiple reaction monitoring mode to monitor mass transitions m/z Q1/Q3 413.45/191.00 (mBB-S-mBB), 447.55/192.00 (mBB-S2-mBB), 477.60/191.00 (mBB-S3-mBB), 509.65/191.00 (mBB-S4-mBB), 543.75/192.00 (mBB-S5-mBB).

### Staining of cells with a polysulfide probe SSP4

The cell staining with SSP4 (Dojindo) was performed with the modified procedure previously described by Chen *et al.*^25^. Cells were washed with DMEM and incubated with 50 μM SSP4 in DMEM containing 0.003% cremophorEL (Sigma-Aldrich) for COS cells or 200 μM CTAB (Sigma-Aldrich) for primary cultures of neurons at 37 °C for 20 min. After washing twice with DMEM, cells were incubated in DMEM containing 500 μM 3 MP for 10 min at 37 °C. The fluorescence images were observed using the confocal laser scanning microscope FV10i (Olympus, Tokyo, Japan).

### PCR analysis

The phenotype of 3MST-KO mice was determined by PCR analysis. Briefly, genomic DNA samples were mixed with forward primers 5′-TTGGTGTGGGATAAGAGACAGG-3′ and 5′-CTTGCAAAATGGCGTTACTTAAGC-3′, and a reverse primer 5′-ACTGTGACAGTATTTCAGGGTAG-3′, and Go Taq Master Mix (Green) (Promega, Madison, WI, USA) to analyze by the procedure recommended by the manufacturer. 3MST knockout genome shows a product with 346 bases and the wild-type genome a product of 578 bases.

### Western blot analysis

Protein samples were analyzed by SDS-PAGE on a 12.5% polyacrylamide gel (DRC, Tokyo, Japan) and transferred to a polyvinylidene difluoride membrane (Millipore, Bedford, MA). The membrane was blocked by PBS-T (137 mM NaCl, 10 mM Na_2_HPO_4_, 2.7 mM KCl, 1.8 mM KH_2_PO_4_, 0.1% Tween 20) containing 2% skim milk (Wako) overnight at 4 °C and incubated with an anti MPST antibody diluted at 1:5,000 for 4 hr at 4 °C. After additional 2 hr incubation with secondary antibody diluted at 1: 20,000 of horse-radish peroxidase-conjugated anti-rabbit (GE Healthcare), the binding of antibodies was visualized with Millipore Immobilon Western Chemiluminescent HRP substrate (Millipore).

### Statistical analysis

All the statistical analyses of the data were performed using Microsoft Excel 2010 for Window 7 (Microsoft, Redmond, WA, USA) with the add-in software Statcel2 (OMS, Saitama, Japan). Differences between 2 groups were analyzed with Student’s *t* test.

## Additional Information

**How to cite this article**: Kimura, Y. *et al.* Identification of H_2_S_3_ and H_2_S produced by 3-mercaptopyruvate sulfurtransferase in the brain. *Sci. Rep.* **5**, 14774; doi: 10.1038/srep14774 (2015).

## Supplementary Material

Supplementary Information
