# Supplementary material for: Identification of H2S3 and H2S produced by 3-mercaptopyruvate sulfurtransferase in the brain
Source: Sci Rep. 2015 Oct 6;5:14774. doi: 10.1038/srep14774 (PMC4594004; doi:10.1038/srep14774)
Supplement: Supplementary Information [file srep14774-s1.pdf]

Supplementary Information

Identification of H_2S_3 and H_2S produced by 3-mercaptopyruvate sulfurtransferase in the brain

Yuka Kimura[1], Yukiko Toyofuku[1], Shin Koike[1, 2], Norihiro Shibuya[1], Noriyuki Nagahara[3], David Lefer[4], Yuki Ogasawara[2], Hideo Kimura[1, *]

1. Department of Molecular Pharmacology, National Institute of Neuroscience, National Center of Neurology and Psychiatry, 4-1-1 Ogawahigashi, Kodaira, Tokyo 187-8502, Japan.
2. Department of Analytical Biochemistry, Meiji Pharmaceutical University, 2-522-1 Noshio, Kiyose, Tokyo 204-8588, Japan.
3. Radioisotope Center, Nippon Medical School, 1-1-5 Sendagi, Bunkyo, Tokyo 113-8602, Japan.
4. Department of Pharmacology and Experimental Therapeutics and Cardiovascular Center of Excellence, LSU Health Science Center, New Orleans, LA 70112, USA.

*Correspondence should be addressed to: Dr. Hideo Kimura, Department of Molecular Pharmacology, National Institute of Neuroscience, National Center of Neurology and Psychiatry, 4-1-1 Ogawahigashi, Kodaira, Tokyo 187-8502, Japan. Tel: +81-42-346-1725, Fax: +81-42-346-1755, E-mail: kimura@ncnp.go.jp.

Figure S1 A representative chromatogram of liquid chromatography with fluorescence detection (LC-FL) and LC-tandem mass spectrometry (LC-MS/MS) of S_1, S_2, S_3, and S_5 monobromobimane adducts. (a) LC-MS/MS (upper 4 columns) and LC-FL chromatograms (bottom column) are shown for S_1, S_2, S_3, and S_5 monobromobimane adducts. Other peaks in LC-FL chromatograms were monobromobimane adducts observed even in the absence of S_n. S_4 monobromobimane adduct was not detected probably due to its instability. (b) Mass spectra for identification of monobromobimane adducts of S_1, S_2, $S_{3,}$ and $S_{5.}$ S_1 and S_3 were analyzed in the negative ion mode, while S_2 and S_5 were analyzed in the positive ion mode.

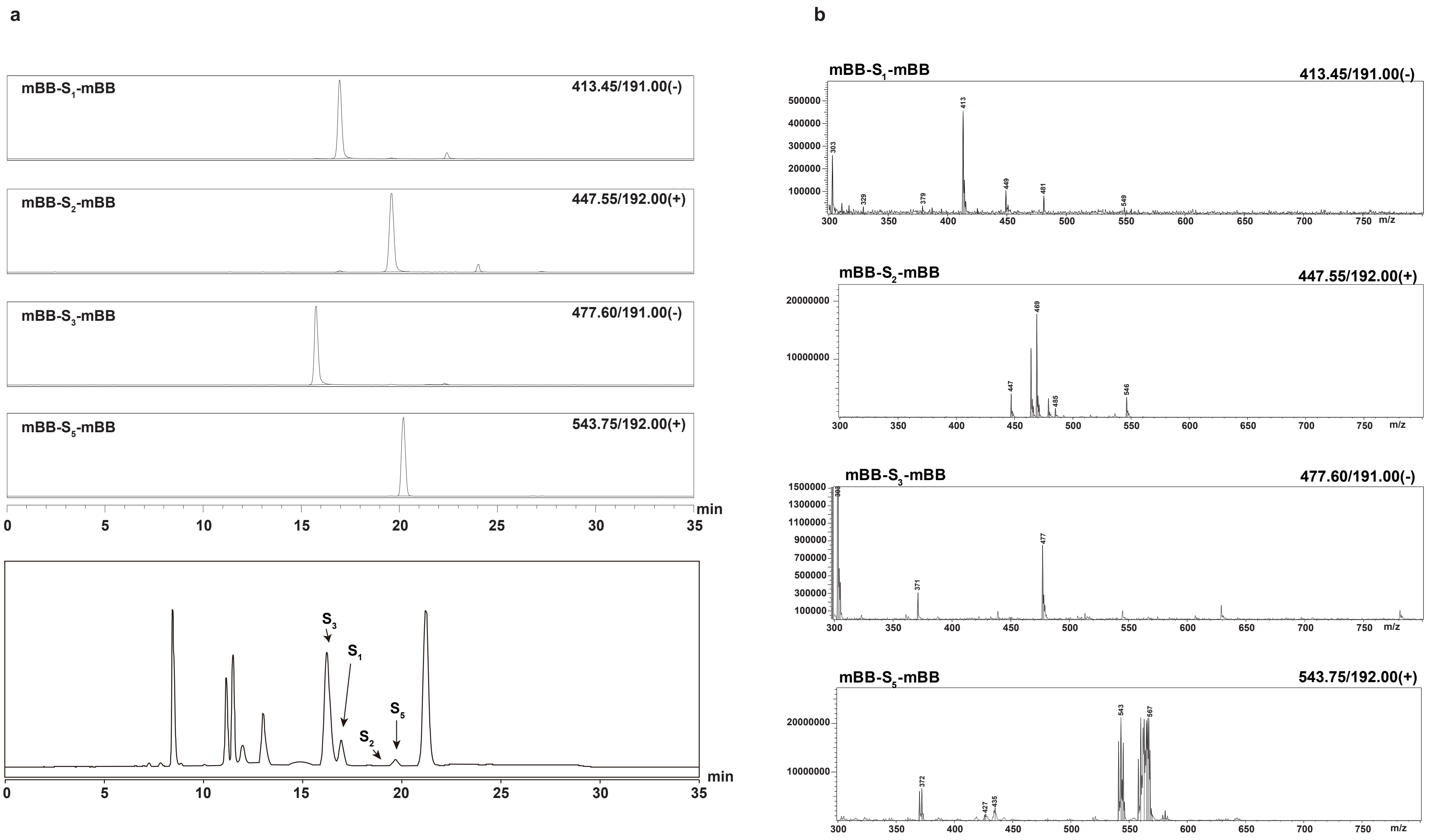
a
mBB-S1-mBB
413.45/191.00(-)
mBB-S2-mBB
447.55/192.00(+)
mBB-S3-mBB
477.60/191.00(-)
mBB-S5-mBB
543.75/192.00(+)
min
S3
S1
S2
S5
b
m/z
